# The EnRiCH Community Resilience Framework for High-Risk Populations

**DOI:** 10.1371/currents.dis.11381147bd5e89e38e78434a732f17db

**Published:** 2014-10-02

**Authors:** Tracey L. O'Sullivan, Craig E. Kuziemsky, Wayne Corneil, Louise Lemyre, Zeno Franco

**Affiliations:** Interdisciplinary School of Health Sciences and Telfer School of Management, University of Ottawa, Ottawa, Ontario, Canada; Telfer School of Management, University of Ottawa, Ottawa, Ontario, Canada; Telfer School of Management, University of Ottawa, Ottawa, Ontario, Canada; School of Psychology, University of Ottawa, Ottawa, Ontario, Canada; Medical College of Wisconsin, Milwaukee, Wisconsin, USA

## Abstract

Introduction: Resilience has been described in many ways and is inherently complex. In essence, it refers to the capacity to face and do well when adversity is encountered. There is a need for empirical research on community level initiatives designed to enhance resilience for high-risk groups as part of an upstream approach to disaster management. In this study, we address this issue, presenting the EnRiCH Community Resilience Framework for High-Risk Populations. Methods: The framework presented in this paper is empirically-based, using qualitative data from focus groups conducted as part of an asset-mapping intervention in five communities in Canada, and builds on extant literature in the fields of disaster and emergency management, health promotion, and community development. Results: Adaptive capacity is placed at the centre of the framework as a focal point, surrounded by four strategic areas for intervention (awareness/communication, asset/resource management, upstream-oriented leadership, and connectedness/engagement). Three drivers of adaptive capacity (empowerment, innovation, and collaboration) cross-cut the strategic areas and represent levers for action which can influence systems, people and institutions through expansion of asset literacy. Each component of the framework is embedded within the complexity and culture of a community. Discussion: We present recommendations for how this framework can be used to guide the design of future resilience-oriented initiatives with particular emphasis on inclusive engagement across a range of functional capabilities.

## Introduction

Definitions of community resilience abound and typically include elements of bouncing back after a traumatic event; adaptive capacity to respond, learn from, and recover from a disruption; and the need to build back better 1 ^,^ 2 ^,^ 3 ^,^ 4 ^,^ 5. But what makes one community resilient and not another? And when a community is not deemed to be resilient, how can it be supported to enhance its adaptive capacity?

In this paper we attempt to provide partial answers to these complex questions. We have previously introduced The EnRiCH Community Intervention as a participatory research initiative focused on enhancing resilience for high-risk populations in five communities in Canada. The first paper in this series highlighted the complexity of factors that contribute to community resilience and critical social infrastructure to promote population health 6. The second paper presented a detailed process evaluation of community consultations used to initiate an asset-mapping task for the five communities, highlighting the experiences of the participants in taking part in these sessions, which were conducted using the Structured Interview Matrix format 7. The process evaluation demonstrated the value of having diverse representation, the importance of empowerment, engagement, and fostering a culture within the group that encourages innovation.

The purpose of this third paper is to expand the discussion on the relevance and importance of empowerment, collaboration and innovation, as core components or drivers supporting adaptive capacity in a community. We present the *EnRiCH Community Resilience Framework for High-Risk Populations*, which describes an integrated, upstream-downstream approach combining principles from health promotion, community-based participatory research, complex adaptive systems, group dynamics, organizational behaviour and disaster management. Following an explanation of the empirical methods and core components of this framework, we present strategic areas for intervention that can be used to enhance resilience amongst high-risk groups in a community. Finally, we discuss broader implications of adopting this asset-based approach for disaster preparedness, both from a policy-perspective, as well as practical aspects of championing this type of participatory approach. However, before introducing the framework, we start by clarifying who we are referring to when we say ‘high-risk populations’ and ‘upstream’ or ‘downstream’ interventions, and what we consider to be key aspects of an asset-approach.

The term ‘vulnerable’ is widely used in the disaster and emergency management literature as a label to describe people with functional limitations who require specific supports when an adverse event occurs. However, Enarson and Walsh^8^ suggested the term ‘high-risk’ be used as an alternative, notably in the context of preparedness and resilience^9^ to move away from deficit-oriented language. This recommendation is based on work by Kailes and Enders^10^ who proposed a function-based approach may be more useful and accurate to identify needed supports during and following a disaster. Informed by their work, we adopted the term ‘high-risk populations’ for The EnRiCH Project and define it as *anyone who has functional limitations related to communication, housing, awareness, mobility/transportation, psychosocial factors, self-care/daily tasks, and safety/security, that may put them at higher risk of negative impacts when an emergency or disaster occurs*. Using this definition, the term ‘high-risk’ can be applied beyond certain groups labeled by age or ability, to include different types of functioning (eg. literacy, activities of daily living, learning capability), as well as the determinants (eg. income, geography, access to information and resources, or chronic and acute medical conditions) which influence functional capabilities. With this in mind, identification of the types of supports needed to support independent functioning during and following a disaster focus on which function is limited, rather than a generalized label^10^ .

In the field of disaster and emergency management, hazard / risk identification, coordinated contingency planning, table top exercises, and disaster drills, are common preparatory activities for building capacity and enhancing community resiliency^11^^,^ 12 ^,^ 13. While these activities are essential for anticipating the ‘what ifs’ and being aware of the risks inherent in a community, most rely on a deficit-based model or lens. Tyler and Moench^5^ refer to this as the ‘predict and prevent’ approach, which is limited by its lack of emphasis on communities’ generalized capacity for adaptive action to unforeseen events.

The upstream–downstream paradigm is often used in the health field to describe preventive and reactive interventions^14^^,^ 16. Downstream refers to the time point when an event occurs and response is necessary, while upstream activities and policy initiatives are put in place to mitigate or reduce negative impacts of a future, potential event. However, in addition to a time-phased relationship, the upstream-downstream paradigm is an integrated, developmental process.

Many communities face challenges with fiscal restraints, competing priorities, and management of multiple risks. Given this context, adaptive downstream response to adverse events requires solid upstream investment toward the development of adaptive capacity. Solid upstream investment in this process requires whole-of-society engagement^17^ , acknowledging the broad influences on health and vulnerability, and encouragement of collaboration and innovative holistic approaches to building resilient communities. The added benefit is a dual investment, which improves the health of the community as a baseline, and contributes to a community’s capacity to adapt in its management of a disaster^18^ .

In their Asset-Based Model for Public Health, Morgan and Ziglio^19^ emphasize the identification of assets for upstream health promotion, while also considering support needs, to address risk factors to well-being. Their model employs a salutogenic foundation (pioneered by Antonovsky)^14^^,^ 20 ^,^ 21, which is oriented toward identifying assets or attributes that contribute to health and resiliency, as opposed to focusing on deficits and vulnerability. An asset-based orientation aligns with a function-based approach^10^ , however in practice many preparedness strategies continue to emphasize deficits and needs, rather than functional capabilities^6^ .

Figure 1 is presented as a generalized map of how the upstream-downstream paradigm integrates with the phases of disaster management in the development and use of adaptive capacity. The four phases, prevention/mitigation, preparedness, response, and recovery^22^ are represented as continuous processes where one phase blends into the next in relation to the occurrence of an adverse event. The prevention/mitigation and preparedness phases are upstream, because developmentally they are oriented toward anticipating future events, taking action toward planning and preparedness, or generalized capacity building. The response and recovery phases occur during and after an adverse event, respectively, and are therefore downstream. In addition to the timeframe, response and recovery activities are reactive, rather than proactive. However, an important developmental transition occurs during the recovery phase. As downstream recovery activities begin to address risk factors and transition into prevention and ‘building back better’, they enhance adaptive capacity for the next event, and therefore become upstream interventions; and the cycle continues. The integration of upstream and downstream strategies is an important paradigm shift in resilience-oriented initiatives. It is a necessary shift to move from silo-ed approaches that divide prevention/mitigation and planning activities from response and recovery, with the recognition of the need to invest in all phases simultaneously. In Figure 1, this continuous, integrated improvement process across all phases is shown as continuous loops in a knot to emphasize the importance of investing in upstream activities that promote adaptive capacity, for downstream benefit when a response is needed.

An additional aspect of this map is the representation of the catalytic roles of empowerment, collaboration and innovation, which we refer to as core drivers of adaptive capacity. During the EnRiCH collaborative asset-mapping activities described in detail below, these drivers emerged as important support mechanisms in the development of adaptive capacity, and are therefore shown in the centre of the knot between upstream investment and downstream adaptive management.

**Generalized map of the development of adaptive capacity:**
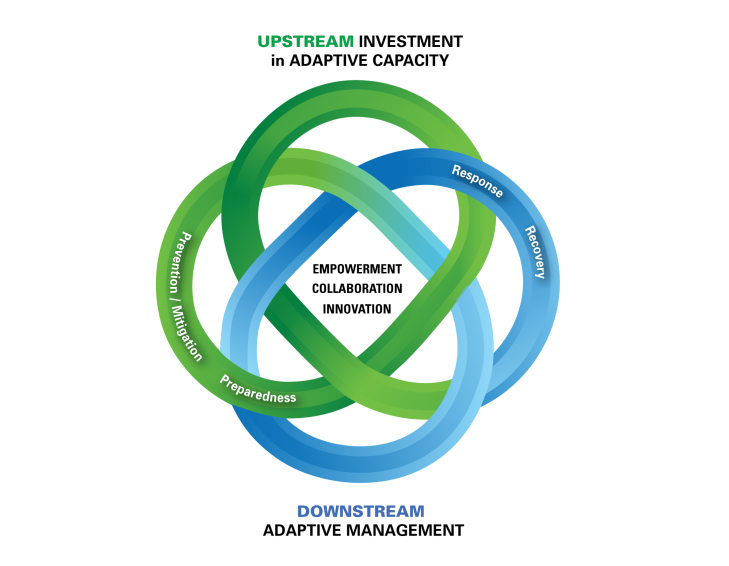

## Method

The EnRiCH Project began in 2009 as a community-based participatory research initiative oriented toward the design, implementation and evaluation of an intervention focused on enhancing community resilience and preparedness for high-risk populations. Following the establishment of partnerships with 5 communities in Canada, and approval from the university research ethics board, an asset/need assessment was conducted in each community using the Structured Interview Matrix (SIM) facilitation technique^7^. Following the asset/need assessment, a collaborative asset-mapping task was completed over an 8-10 week period in each community. Each component is described in detail within the EnRiCH Community Intervention Manual^23^ which is available on the project website (www.enrichproject.ca), and a brief summary is provided below.

Before data collection for each component of the project, all participants were provided with a detailed description of the data collection and management protocols. Each participant signed a consent form approved by the university research ethics board. For the asset/need assessment component, 9 consultation sessions were held across the 5 geographically defined communities, with (*n*=143) participants recruited from diverse sectors, such as emergency management, health and social services, and community support associations. Each SIM session was facilitated in a series of 3 steps: 1) One to one interviews among the participants; 2) small group deliberation; and 3) plenary group discussion. During each 4.5-hour session, 8 questions were addressed, exploring the strengths, weaknesses, opportunities and threats for community preparedness and well-being of high-risk populations. Each session was audio-recorded, transcribed verbatim, and checked for accuracy, in preparation for content analysis. A full explanation of the SIM technique and the asset/need assessment phase is described in the intervention manual^23^.

The collaborative asset-mapping task was conducted in 4 of the EnRiCH communities, with the objective of enhancing awareness, relationships, and inclusive engagement among diverse groups within the community. The 5^th^ community did not participate in this phase of the project due to extensive restructuring of the organization that was leading this initiative. The asset-mapping component of the study was implemented in 3 phases: 1) A full day session where participants were informed about the asset-mapping process and how to use an online collaborative tool; 2) a 8-10 week period where the group worked collaboratively to complete the asset-mapping task; and 3) a follow up table top session to assess the utility and appropriateness of using the asset-map for community response to a disaster^23^. The table top exercise was tailored for each community, however the template was a disaster scenario involving a train derailment where toxic chemicals threatened the health of the population. It was a complex scenario given the incident was situated in a neighourhood where there was a high proportion of people with functional limitations that would require unique supports during evacuation. The asset-mapping and table-top sessions were all audio-recorded, transcribed verbatim, and checked for accuracy.

Analysis for this study began with the development of a preliminary framework, based on themes from the asset/need assessment data. We identified broad categories from the emergent themes, and integrated them with concepts from extant literature on resilience, emergency management, community engagement, interdisciplinary collaboration, and the use of a function-based approach. The work of Israel et al.^24^, Kailes and Enders^10^ , and Norris et al.^4^ were particularly influential as we worked through different iterations of this framework to represent the themes that emerged from our analysis of the transcripts from the EnRiCH SIM sessions. The principles of community-based participatory research^24^ provided the foundation for the establishment of partnerships and ensuring diverse participation and local leadership, while the function-based approach described by Kailes and Enders^10^ formed the basis for our strategies to promote inclusive engagement. The strategic areas for intervention described below, are an expansion of the four types of adaptive capacities presented by Norris et al.^4^.

To ensure rigour in the development and revision of the framework, the preliminary categories were used to code 2 transcripts from the asset-mapping phase. Using a consensus approach, over several meetings, the research team revised the framework to ensure it accurately represented the EnRiCH collaboration in each community. The revised framework is presented below. As shown in Figure 1, the integration of upstream and downstream strategies is a continuous cycle as one phase of a disaster blends into another. Figure 2 is presented as a more detailed account of this integration.

## EnRiCH Community Resilience Framework for High-Risk Populations

Adaptive capacity, which is a dynamic state, is placed at the centre of the framework, as the foundation of a resilient community. Empowerment, innovation and collaboration (shown in the inner ring) are core drivers of adaptive capacity; while upstream-oriented leadership, asset/resource management, awareness/communication, and connectedness/engagement (shown as four quadrants), are strategic areas for intervention, building on the key adaptive capacities outlined by Norris et al.^4^. All the components are embedded within the culture of a community, shown as the outermost ring in the lens. This orientation underscores the need for appropriate, feasible, and tailored strategies that align with the context of the specific community.

Complexity, also placed in the outer ring of the diagram, is an integral aspect of the framework and must be considered in the design, implementation and evaluation of any resilience-oriented intervention^6^. The tenets of complexity, such as self-organization, non-linearity, emergent behaviors, interconnectivity, and feedback influence each component of the framework. Dynamic context, another tenet of complexity, is inherent across all socio-ecological levels, and is therefore an underlying assumption.

**The EnRiCH Community Resilience Framework for High-Risk Populations (Adapted from Norris et al., 2008):**
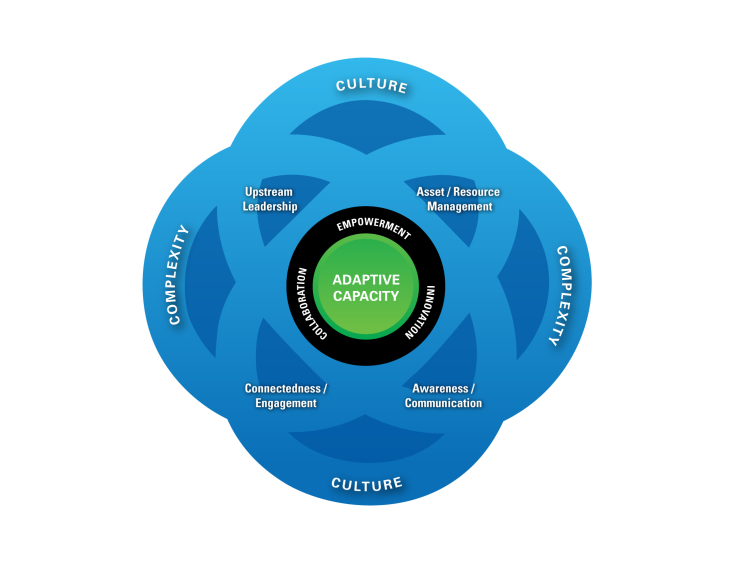

Drivers of Adaptive Capacity

As shown in Figure 1, the enhancement of adaptive capacity within a community is a developmental process stemming from upstream investment in prevention/mitigation and preparedness activities, to maximize adaptive capacity for downstream response and recovery when a disaster occurs. While the term resilience comes from the concept of strength, in an integrated paradigm, it is discussed in terms of flexibility to changing context, learning and adaptation^25^^,^ 5. Tyler and Moench^5^ provide a clear distinction between robust and resilient systems. They describe robust systems as focused on strength, and avoiding failure to maintain function. In contrast, resilient systems focus on flexibility and diversification of functional dependence, to ensure function is maintained if different components of the system fail. This concept is particularly relevant for disasters, given that some systems go beyond bending and actually break; creating a need for repair and reconfiguration. Adaptive capacity is central to this distinction as it underscores the ability of a system to respond to uncertainty in innovative ways, rather than ‘holding strong’ as the ultimate outcome.

Norris et al.^4^ describe resilience as a set of adaptive capacities. When a system is resilient, it is able to respond to changing context and remain functional. Ultimately it will recover from the required adaptation with improved capacity or operational protocols. For communities, organizations, or individuals faced with a crisis, an ideal outcome is to adapt and respond to the changing context, while remaining healthy and functional, as well as recover in a way that contributes to further capacity for future well-being. In the EnRiCH framework presented here, adaptive capacity is shown as the intended outcome. It is driven by *empowerment*, *collaboration*, and *innovation* across four strategic areas for intervention (upstream-oriented leadership, asset/resource management, awareness/communication, and connectedness/engagement). These strategic areas of intervention are relevant for all phases of disasters, as a cyclical process, shown above in Figure 1.

Adaptive capacity, while complex, can be identified through indicators such as functioning, health status, skills, and assets/resources^4^. Adaptive management builds on adaptive capacity and incorporates these indicators; but it is also facilitated by empowerment of different people and organizations within a community, collaborative practice and innovative activation of assets to meet changing demands^5^^,^ 11 ^,^ 26. There is a substantial body of literature highlighting the importance of acknowledging and embracing innovation and emergence as natural and beneficial attributes inherent in the complexity of disasters^25^. When new behaviours or practices emerge, it provides opportunities to see many ways systems can be configured – or reconfigured as the case may be. This acceptance and openness to possibilities that arise when emergence occurs is part of a culture that encourages innovation and empowers the community to contribute^25^.

Collaboration, the third driver of adaptive capacity, enables activation of assets through social networks and is an interpersonal and organizational capability. There is prolific literature suggesting collaboration should be emphasized through all phases of disaster management as a continuous process^27^^,^ 28, and the findings from EnRiCH support this recommendation^29^. Collaboration is dynamic, emergent, and moves through several stages: 1) Coordination, 2) cooperation, and finally 3) collaboration^31^^,^ 32. Elmarvsouqi et al.^31^ provide a good explanation of how collaborative relationships evolve and move beyond coordination and cooperation, toward collaboration. However, what is essential in this evolution is the investment of time and energy to build awareness and common ground^33^; common ground referring to shared beliefs and understanding between members of a collaborative group or team 34 ^,^ 35.

Organizations can prioritize and invest in collaboration by emphasizing its importance in strategic plans, job descriptions, and leadership training, while ensuring appropriate resources are allocated to allow people to invest time in developing relationships. Intervention strategies to promote collaborative practice among agents in a community can be targeted at the micro level (between individuals), meso level (between organizations), or at the macro level (between communities). An empowering learning culture, which supports and encourages innovation, is an essential ingredient in the process of collaborative practice, system change 36 ^,^ 13 ^,^ 37 and activating assets in any adaptive response^6^ .

The core drivers of adaptive capacity cross-cut each strategic areas for intervention in this framework. Each strategic area will be discussed in terms of how it contributes to resilience, and how empowerment, innovation and collaboration can be facilitated through a participatory approach, to enhance adaptive capacity, while acknowledging the complexity and uniqueness of culture in each community.

Awareness / Communication

In promoting community resilience, it is essential to understand the information individuals and organizations use, and how it is exchanged to support awareness, communication, and to transform behaviour. Information exchange is complex, and it involves the push and pull of information across micro, meso and macro levels. It is influenced by the timing, volume and appropriateness of the content to meet stakeholder needs. An essential process in awareness-building activities and collaborative practice, information exchange helps refine or alter the lens through which people see the assets in their community. It also helps people understand the context of a given situation, and trigger empathy for the challenges other people face in their jobs and everyday lives. As described by one participant in the EnRiCH sessions,

“when I came today I wasn’t sure what to expect. Not only have I learned what is available in terms of resources/assets, but it has also made me query the definite weaknesses we have in [the community]. My thoughts are to share the info I have with co-workers and because of the extreme disaster in Japan, to focus more on preparedness for individuals with disabilities.”

Awareness is dynamic, and it is fostered through exchange of information, collaborative learning, and engagement 5 ^,^ 13 ^,^ 33 ^,^ 35. Adaptive action emerges from accessing and understanding information, and knowing how to apply that information for innovative response. Individual and collective awareness are assets themselves; in fact Berkes 26 suggests that basic literacy is one of the foundational assets for resilience in a community, and contributes to awareness. This is consistent with extant knowledge of the social determinants of health, suggesting that upstream efforts to improve literacy contribute to many of the other determinants that support population health 16.

Evidence from the EnRiCH community intervention suggests adaptive capacity and action are determined by individual and collective *asset literacy*, which we have previously described as a type of awareness 7. However, our understanding of asset literacy is evolving and we now regard it as a capacity which moves people and organizations from awareness to action, and define it here as *an understanding of what assets are, their value-add, how to mobilize them, and a willingness to active them. *Empowerment, facilitated through inclusive engagement and openness to innovation, is essential for supporting asset literacy within a community.

It is essential to recognize how asset literacy contributes to adaptive management, and to design intervention activities to expand it, so people understand how different assets may can be combined into new configurations that address situational demands, including but not limited to those of crisis. Activities to enhance asset literacy align with community-based participatory principles of building on the existing strengths of a community 24. Taking time to listen and learn about people, including their background, interests, experiences, and the activities they participate in, is part of developing partnerships based on mutual respect. It can be as simple as going for coffee and learning about what ‘makes people tick’, or letting people know that their experience or expertise is important and appreciated. This conversation is the process whereby people learn about each others’ assets as relationships evolve; people also learn about their own assets by how others’ value them.

Role modeling of empowering language is an important aspect of effective communication, particularly in interventions designed to enhance awareness and asset literacy. Language can determine whether people see attributes as assets or deficits, and contributes to attitudes oriented toward functioning or limitations. A prominent recommendation in disaster management is to ensure ‘vulnerable populations’ are protected; however, moving toward an asset-based lens, requires a shift in awareness and language. One practical strategy to make this shift is to discard the term ‘vulnerable populations’. In this paper and throughout The EnRiCH Project we have used the term ‘high-risk populations’ as a substitute for ‘vulnerable populations’, based on recommendations by Enarson and Walsh^8^ and Lemyre et al.^9^ . The label ‘vulnerable’ is deficit-oriented and focuses on need and dependency. It implies an individual has little to contribute and is likely to consume resources. Alternatively, the term ‘high-risk’ takes the emphasis away from the individual, and focuses on the changing context.

A second example of how language can be used as a participatory strategy to support asset literacy is making a distinction between the terms ‘functional need’ and ‘functional capability’. The former puts the emphasis on needs and deficits, whereas the latter focuses on what an individual is capable of doing, with or without support. During the EnRiCH consultations it became apparent that typically when a group is asked about needs, the discussion will focus on gaps. However, when a group is asked about assets, the discussion provides a more balanced representation of both assets and gaps. In other words, by default the discussion often turns to what is missing. Therefore, when employing an asset-based approach, it is important to facilitate the discussion so an accurate asset map can be determined. This distinction is fundamental in the development of asset literacy.

We recognize that language is evolutionary and represents the dominant paradigm at a given time, therefore the terms used to describe different groups within the population need to be gradually shifted to keep pace with societal change. This type of shift has profound implications for how a community approaches its sustainable development. When people are regarded first as assets, it opens the possibility for identifying different resources and contributions they can provide. Labels are an important aspect of changing the lens in a community, and a whole-of-society approach moves the yardstick to view all members in a community as assets and to consider their potential contributions.

Empowerment can be fostered through activities oriented toward developing awareness about language and enhancing common ground^6^ . In doing so, it is important to ask reflective questions about how different groups are viewed in a community. ‘*How are the tasks described*?’ ‘*Are objectives described in terms of protecting people, addressing vulnerabilities or promoting development and building on assets*?’ *‘Does the language referring to different populations focus on ‘us’ or ‘them*’?’. Another question to ask is *whether functional capabilities are recognized in everyone, or are people defined according to their limitations, using deficit labels (eg. wheelchair users; mentally ill; frail elderly)*?’ Collective asset literacy can be enhanced through group consultations to address these questions and establish common ground across different sectors. The expertise of community groups is an important asset in this respect, and can be used in collaborative practice, training, policy review, and discussions around preparedness. The methods used to conduct these types of activities are described in the EnRiCH Community Intervention Manual^23^ .

Another important consideration related to awareness/communication, as a strategic area of intervention, is to understand how information is exchanged within and between communities, paying particular attention to how culture and context influence sociotechnical aspects of how information sharing^37^. An important step in designing resilience interventions is to examine what information systems are already in place, and to explore windows of opportunity where innovation can improve adaptive capacity. This practice provides insight about how assets can be activated or refined to address emergent needs and promote collaborative practice. During these discussions it is important to establish an empowering atmosphere where people are invited to express their opinions and ask questions. It is also important for planners and facilitators to maintain an open mindset to consider innovative solutions that may challenge the status quo; sometimes what emerges are simpler solutions.

Asset/Resource Management

Each person, household, and organization within a community has a unique set of assets that can be activated in a disaster response. This set is what we refer to as an *asset profile*. Assets are used every day in activities of daily living, yet people may not recognize their skills or other attributes as assets. Furthermore, asset profiles are dynamic; as people learn and interact with other people, organizations and their environment, they develop, enhance, or in some cases depreciate, their assets.

Moser and Satterthwaite^38^ identified different categories of assets, such as financial, physical, natural, social, and human (eg. health, skills, knowledge). A functional capabilities approach focuses on assets across a range of categories that contribute to functional independence^23^. This approach aligns with the paradigm of person-centred care, where health care providers and others in society are encouraged to see the person before the disability or other type of functional limitation^39^. Adoption of this approach in disaster and emergency management provides a context for seeing the potential contribution people can make in building a more resilient community, rather than emphasizing the resources they may consume when an adverse event occurs.

The need to recognize asset profiles of people within the community was highlighted in an interview with a participant who stated, “*no one asks people with disabilities to volunteer … they assume we can’t do things*”. This same individual was interested in sharing her expertise about computers and technology, and was enthusiastic to get involved and build social connections. Through the connections she made while participating in the EnRiCH asset-mapping sessions, she was invited to teach computer classes at the local library. When reflecting on this, she described how her confidence had increased, she made friends, and she was pleased to be making a contribution to her community. Through encouragement and having opportunities to engage, she activated and enhanced several types of assets within her profile (eg. confidence, knowledge, social capital), and the community benefitted from her expertise and willingness to share her knowledge of computers with others. In essence there was an expansion of her personal asset literacy and the collective asset literacy of her community.

Mapping of assets is a community development strategy^40^ and an important upstream investment for disaster management. While Moser and Satterthwaite^38^ categorized assets for individuals, households, and communities, it is important to keep in mind that strengthening of asset profiles of people and households feeds forward to enhance the asset profiles of organizations, because people act on and manage the systems and institutions^5^. Innovation can be fostered through asset-mapping interventions that empower organizations which normally do not have opportunities to work collaboratively, creating opportunities for co-learning, enhancing awareness, and prompting new ways of thinking^7^^,^ 13.

Comprehensive identification of assets requires: 1) A diverse and creative participant mix for asset-mapping exercises, 2) investment of resources to facilitate involvement, 3) out-of-the-box strategies to ensure good exchange of information and effective communication, and 4) facilitation that enables true engagement. The innovation and self-organization that comes from the sharing of diverse expertise provides benefits not only for the community or organization as a whole, but also for the expansion of asset literacy. Collaborative groups can also focus on developing additional assets to address functional capabilities and needs across a variety of contexts. When a crisis or disaster occurs, prior upstream investments in mapping and articulating connections between assets in the community can be used for rapid, adaptive downstream response.

Upstream-Oriented Leadership

Coiera^37^ describes the need for complex systems to overcome inertia and resistance to change. Allocation of resources toward upstream initiatives is a fundamental requirement where asset/resource management and leadership intersect in this framework for community resilience. Hobfoll^41^ describes community capacity in terms of resource loss and gain. When communities have adequate assets/resources to meet the demands imposed by an event, they are able to adapt to the changing context and either resist, absorb, or adjust as needed^3^. Upstream investment requires resources such as time, energy and money in the identification and building of asset profiles. Investment in innovative solutions to reconfigure systems and/or protocols allows communities to overcome inertia and enhance adaptive capacity for downstream response.

The upstream-downstream paradigm oriented toward sustainable community development is important for facilitating adaptive management^5^^,^ 26, and recognizes the need to strengthen asset profiles and contribute to healthy, vibrant communities ahead of a disaster^18^. The result is a doubling-up of investments, where innovative interventions to solve downstream response concerns also strengthen ongoing quality of life in a sustainable way. For example, investments in universally designed recreation centres provide daily access to facilities which promote empowerment, social engagement and physical activity, while at the same time investing in suitable shelters that can be used in downstream response to address the needs of the whole community, particularly people with functional limitations.

It is a challenge to motivate people and organizations to prepare for disasters^42^, particularly in areas where adverse events are rare. People are often motivated after an event occurs (either in their community or elsewhere), but an ongoing challenge for emergency planners is to maintain that motivation after the event leaves the spotlight. Upstream initiatives are paramount to building adaptive capacity for all types of adverse events, and they require effort, investment, creativity, and motivation in non-disaster times.

An indication of whether a community has adopted the integrated upstream-downstream paradigm is how the collective vision and investment priorities address social determinants of health, which contribute to community resilience^16^. For example, distribution of income within a community, employment opportunities, education and literacy, accessibility of health services, inclusive orientation of policy development, and social networks all determine asset profiles, and influence peoples’ capacity to adapt to adverse events. The determinants tend to cluster, as they are interconnected and feed forward as complex influences on health and functioning; people who experience disadvantages because of one determinant (eg. unemployment) are often at-risk in one of the other determinants as well^16^ .

An integrated upstream-downstream approach requires championing. It requires innovative activities related to each of the strategic areas for intervention outlined in this framework, and within each activity fostering empowerment, innovation and collaborative practice. Creativity is a precursor of innovation, but must be activated through a culture that encourages innovation and empowerment^6^^,^ 43. Transformational leadership is needed to foster this type of culture; communicating a vision and empowering others to help shape and refine it. Recognition of individual expertise (an important aspect of an individual’s asset profile) and their potential contribution toward that shared vision is a defining feature of this style. Pertinent questions to ask when exploring whether a community or organization is oriented toward upstream leadership include: ‘*What lens do leaders use when approaching disaster management*?’ ‘*Are they focused on vulnerabilities or assets (or both in combination)*?’ ‘*Do response organizations view their roles as protection of high-risk populations, or as a collaborative endeavor with members of high-risk populations and the community associations which support them*?’ Answers to these questions provide clues to where interventions can be targeted for maximum return on investment.

Connectedness / Engagement

The fourth area for strategic intervention in this framework is connectedness/engagement, which focuses on reaching out to people and organizations to establish networks and relationships, enhance asset literacy, and provide genuine opportunities for participation. Social networks are recognized as important assets and their potential has increased through advances in social media (eg. Twitter and LinkedIn)^44^. While the benefits of some connections are not always obvious, they may contribute to the asset profiles of individuals, households and organizations, by providing a link to information and opportunities^45^. These networks also represent important communication channels for enhancing awareness, messaging, and stimulating social change (for example, motivation toward household preparedness).

Social environments are important determinants of health^16^. When people interact with others and have a sense of belonging, it enhances psychological well-being, and contributes to self-determination and overall health^46^. Opportunities for engagement contribute not only to asset literacy of the individuals involved, but they benefit the organizations in which these people are based. In turn, the community benefits from a more engaged, healthier population, with social resources and social infrastructure to support daily activities, enhancing capacity for adaptive management when adverse events occur^4^.

Diverse participation in the asset-mapping intervention for EnRiCH was an important element of fostering connectedness and engagement. Recruitment was intentionally broad, to invite people with varied backgrounds and expertise to contribute. During the recruitment process, some organizations expressed uncertainty about whether they had expertise that would be beneficial to the process. Through an explanation of what assets are and why the asset-mapping exercise was being done, they understood that their knowledge/expertise was indeed an asset that could be activated within the community^7^. The experience of one participant was expressed at the end of the SIM session in her community: *“We covered a lot of ground. The format allowed personal contacts and sharing of information. I’m hearing that others, like myself, are feeling more capable and empowered to go forward and work on disaster planning.”*

In this type of asset-mapping intervention, it is essential for leaders to be visible and model the type of behavior needed for inclusive engagement, communicating the message that each person in the group is a valued participant. Efforts to greet each person and allowing time for each person to be introduced are positive strategies to be inclusive and appreciate peoples’ time. Other strategies include adapting sessions to accommodate different functional limitations, so everyone can participate and truly engage in the discussion. These efforts not only empower participants with functional limitations, but they provide role modeling for other participants, showing that it is important to take the time to converse with everyone, particularly people who may need to speak slowly or use alternate methods to communicate.

A fundamental principle being emphasized in this part of the framework is that the invitation to participate in the asset-mapping discussions opens the door to engage the community, but it is important to take the next step and ensure everyone is provided with appropriate supports to participate when they are there. This involves planning strategies in the facilitation of the discussion to ensure all opinions in the room can be voiced, and people feel empowered to deliberate during the discussion. These are important aspects of building common ground, enhancing asset literacy, and supporting innovation.

## Implications

This EnRiCH framework provides a structure that communities can use to target upstream investment activities, by promoting a culture of empowerment, innovation, and collaboration across the strategic areas of intervention (awareness/communication, asset/resource management, upstream-oriented leadership, and connectedness/engagement). Inclusive engagement and collaborative practice with diverse community groups are important elements of an integrated, upstream-downstream approach to enhancing adaptive capacity, and hence resilience, among high-risk populations. They also align with a human rights approach to disaster risk reduction. Investment in activities to engage people and organizations in asset-mapping is a good starting point for understanding attributes in a community that contribute to resilience^11^^,^ 29 ^,^ 40 ^,^ 47.

The impact of a disaster is situated in the context of a community at any point in time. However, in recent years there has been more emphasis on the need to measure resilience and develop appropriate and diverse indicators to quantify how ‘ready’ a community is to respond and recover from any adverse event it encounters. Measurement of resilience is a challenge, because a community can only demonstrate resilience in response to an adverse event. However the identification of the core drivers of adaptive capacity provides building blocks that can be used to develop indicators to measure resilience throughout phases of a disaster, recognizing the integrated upstream-downstream cycle. Evidence from this EnRiCH community intervention suggests that asset literacy, perceptions of connectedness, openness to innovation, and common ground are potential indicators that could be developed to measure adaptive capacity. Future research could focus on developing indicators for each of the core drivers of adaptive capacity and strategic areas of intervention presented in this framework.

In this framework, we do not address how resilience influences economic development. This is an important area of future research to demonstrate how upstream community investment contributes to downstream economic benefits. The influence of different types of emergence and how they influence recovery and upstream efforts to plan for the next event could be explored using sensitivity analyses. Additional areas of exploration could focus on how this framework generalizes to other locations and countries, and whether automated or technology-based asset-mapping can facilitate expansion of personal and collective asset literacy. It will also be important to understand the realities and limits of partnership with community organizations using this approach, and explore strategies to facilitate institutionalization of these resilience concepts by different types of organizations as they engage in continuity of operations planning.
